# Curcumin combined with metformin decreases glycemia and dyslipidemia, and increases paraoxonase activity in diabetic rats

**DOI:** 10.1186/s13098-019-0431-0

**Published:** 2019-04-30

**Authors:** Daniela Fernandes Roxo, Carlos Alberto Arcaro, Vania Ortega Gutierres, Mariana Campos Costa, Juliana Oriel Oliveira, Tayra Ferreira Oliveira Lima, Renata Pires Assis, Iguatemy Lourenço Brunetti, Amanda Martins Baviera

**Affiliations:** 1 São Paulo State University (Unesp), School of Pharmaceutical Sciences, Department of Clinical Analysis, Rodovia Araraquara Jaú, km 01-s/n, Campos Ville, Araraquara, São Paulo 14800-903 Brazil; 2 Paulista University (UNIP), Institute of Health Sciences, Avenida Alberto Benassi, 200, Araraquara, 14804-300 São Paulo Brazil

**Keywords:** Diabetes mellitus, Glycoxidative stress, Paraoxonase, Curcumin

## Abstract

**Background:**

Combination of current antidiabetic agents with natural antioxidants to manage diabetes mellitus and its complications has appeared as an emerging trend. Curcumin, a yellow pigment isolated from *Curcuma longa* rhizomes, has gained attention due to its beneficial effects in controlling the disturbances observed in diabetes mellitus. The purpose of this study was to investigate if yoghurt enriched with curcumin and metformin, individually or as mixtures, ameliorates physiometabolic parameters, glycoxidative stress biomarkers, and paraoxonase 1 (PON 1) activity in diabetic rats.

**Methods:**

Streptozotocin-diabetic rats (6-week-old Wistar rats) were treated for 30 days with curcumin and metformin, isolated or as mixtures in yoghurt (10 rats/group). After treatments, the plasma levels of glucose, triacylglycerol, cholesterol, thiobarbituric acid reactive substances (TBARS, a biomarker of lipid oxidation), fluorescent advanced glycation end products (AGEs), and the activity of PON 1, an antioxidant enzyme were assessed. Data were analyzed using one-way analysis of variance (ANOVA) followed by Student–Newman–Keuls test.

**Results:**

Treatment of diabetic rats with curcumin or metformin alone decreased the plasma levels of glucose, triacylglycerol, cholesterol, TBARS, and fluorescent AGEs, as well as increased the activity of PON 1. The combination of metformin with curcumin further decreased dyslipidemia and TBARS levels in diabetic rats, indicating synergy, and maintained the high levels of PON 1.

**Conclusion:**

These findings indicated that curcumin combined with metformin may act synergistically on dyslipidemia and oxidative stress, as well as increased PON 1 levels. Therefore, it might be a promising strategy for combating diabetic complications, mainly the cardiovascular events.

## Background

Diabetes mellitus (DM) is a chronic metabolic syndrome resulting from defects in pancreatic insulin secretion and/or insulin action on target tissues. The increased rates of mortality and morbidity related to DM are often associated with macro- and microvascular complications. Persistent hyperglycemia participates in the onset and maintenance of these complications, mainly via oxidative stress [1]. In spite of the availability of various antidiabetic agents controlling hyperglycemia, therapeutic options targeting other disturbances often related to DM, such as dyslipidemia and oxidative stress have also been a major focus in research. Therefore, it is crucial to explore new agents and strategies as the epidemic proportions of DM continue to increase. Combination of the actual available antidiabetic drugs with phytochemicals has appeared as an interesting strategy to manage hyperglycemia and other DM disorders. Curcumin (diferuloylmethane) is a yellow pigment isolated from dried rhizomes of *Curcuma longa* L. (turmeric), which is largely used as a dietary spice. Focusing on diabetes, curcumin has gained attention due to its ability to ameliorate hyperglycemia and exert a range of beneficial effects on macro- and microvascular complications, including cardiovascular diseases [2], nephropathy [3], retinopathy [4], and endothelial dysfunction [5]. Furthermore, studies are showing promising findings on the efficacy of curcumin in combination with antidiabetic agents [6] or with other phytochemicals [7] in the control of glycemia and attenuating other diabetic complications.

The purpose of this study was to investigate the effects of curcumin combined with metformin on the changes in the levels of biomarkers of metabolic disturbances, oxidative stress, and antioxidant defenses in the plasma of streptozotocin-diabetic rats.

## Methods

### Animals and induction of diabetes mellitus

Male Wistar rats (*Rattus norvegicus*; 6-week-old; weighing 140–160 g) were housed in individual metabolic cages under controlled conditions of temperature (23 ± 1 °C) and humidity (55 ± 5%) and a 12 h light/dark cycle. Rats received water and lab chow diet (Presence Nutrição Animal, Paulínia, São Paulo, Brazil) ad libitum throughout the 30 days of the experiment.

After an initial period of adaptation, experimental type 1 DM was induced with a single intravenous injection of 40 mg/kg streptozotocin (STZ) (Sigma Aldrich, St. Louis, Missouri, USA) dissolved in 0.01 M citrate buffer (pH 4.5) in rats subjected to 14 h fasting. Normal rats received only citrate buffer. For this procedure, all the animals were anesthetized using isoflurane. Three days after STZ administration, diabetic rats were divided into the different experimental groups according to similar medium values of glycemia and body weight, taking into account that the glycemia levels before treatment ranged between 380 and 510 mg/dL. All the experimental groups having diabetic rats started with similar medium values of glycemia (≈ 400 mg/dL), to avoid bias, and effectively interpret the changes observed in glycemia levels resulting from the treatments throughout the 30 days of the experiment.

### Experimental design and treatments

Curcumin from *Curcuma longa* (Turmeric; purity of 76%; product number C1386, batch number SLBH2403V; purchased from Sigma Aldrich, St. Louis, Missouri, USA) and metformin (metformin hydrochloride; purity of 99.56%; purchased from Gemini Indústria de Insumos Farmacêuticos Ltda, Anapolis, Goias, Brazil) were mixed with commercial plain yoghurt (170 g containing 9.1 g carbohydrates, 6.8 g protein, 7.0 g total fat, 126 kcal, Nestlé^®^, Brazil) using a homogenizer (27,000 rpm) for 90 s at ambient temperature (25 °C).

Diabetic rats were distributed into 5 groups (10 rats/group): diabetic rats treated with yoghurt (DYOG), diabetic rats treated with 90 mg/kg curcumin in yoghurt (DC_90_), diabetic rats treated with 250 mg/kg metformin in yoghurt (DM_250_), diabetic rats treated with 90 mg/kg curcumin + 250 mg/kg metformin in yoghurt (DC_90_M_250_), diabetic rats treated with 4 U/day insulin (DINS). The experiment also had a group of normal rats treated with yoghurt (NYOG).

The oral treatments were given by gavage twice a day. Curcumin and/or metformin were administered as a half dose (45 mg/kg/gavage for curcumin and 125 mg/kg/gavage for metformin) in 0.5 mL of yoghurt, totaling 1.0 mL/rat/day of treatments. Insulin treatments were also given twice a day; rats received 2 subcutaneous injections of insulin (Biohulin^®^, NU-100, Brazil), 2 U/rat each injection at 08:00 h and 17:00 h for 30 days.

During the experiment, body weight, food and water intake, and urinary volume were monitored over a period of 24 h every week. An aliquot of urine was taken to determine glycosuria levels (o-toluidine method). The blood samples were collected weekly, from the tip of the tail, in heparinized tubes (Hemofol^®^, 5000 UI/mL), and the plasma samples were used to determine glycemia levels, using commercial kit (Labtest Diagnostica SA, Lagoa Santa, Minas Gerais, Brazil).

At the end of the treatments, the blood samples were collected for the analysis of plasma levels of glucose, triacylglycerol, cholesterol (Labtest Diagnostica SA, Lagoa Santa, Minas Gerais, Brazil), and for the measurement of thiobarbituric acid reactive substances (TBARS), fluorescent advanced glycation end products (AGEs), and the activity of the antioxidant enzyme paraoxonase 1 (PON 1).

The experimental procedures were approved by the Committee for Ethics in Animal Experimentation from the School of Pharmaceutical Sciences, São Paulo State University (UNESP), Araraquara, SP, Brazil (CEUA/FCF/CAr resolution number 23/2017).

#### Thiobarbituric acid reactive substances (TBARS)

Lipid peroxidation products, including malondialdehyde, were measured in deproteinized plasma samples using the thiobarbituric acid (TBA) reaction [8]. TBA reactive substances (TBARS) were measured fluorometrically with excitation and emission wavelengths of 510 and 553 nm, respectively. We used 1,1,3,3-tetramethoxypropane (Sigma Aldrich, St. Louis, Missouri, USA) as standard. The results were expressed as µmol/L.

#### Fluorescent advanced glycation end products

The fluorescence relative to advanced glycation end products (AGEs) was determined according to Zilin et al. [9], with some modifications. To plasma samples were added 1.2 M chloroform, 0.12 M trichloroacetic acid, and 0.1 M sodium hydroxide. The tubes were shaken vigorously and maintained at 10 °C ± 2 °C for 30 min, and then were centrifuged at 10,000*g* for 10 min at 10 °C. Supernatants were used to measure AGEs spectrofluorometrically, with excitation and emission wavelengths of 370 and 440 nm, respectively, using a microplate multi-mode reader, with split set at 16 nm, Synergy H1™, BioTek Instruments Inc (Winooski, VT, USA). The results were expressed as arbitrary units of fluorescence.

#### Paraoxonase 1 (PON 1) activity

PON 1 activity was determined according to Assis et al. [7], via hydrolysis of paraoxon and the release of *p*-nitrophenol. The activity was monitored by measuring the absorbance at 405 nm over a period of 5 min. The results were calculated using the molar extinction coefficient of *p*-nitrophenol (18,000/M/cm). PON 1 activity was expressed in units/liter (unit = μmoL paraoxon hydrolyzed/min).

### Statistical analysis

Data were expressed as mean ± standard error of mean (SEM). One-way analysis of variance (ANOVA) followed by the Student–Newman–Keuls test were used to compare the temporal inter-group differences in physiological and biochemical parameters as well as in the glycoxidative stress biomarkers (TBARS, AGEs, PON 1). Paired Student’s *t* test was used to compare intra-group changes in parameters relative to day 0. Data were considered statistically significant at p < 0.05. Statistical analyses were performed using the program Graphpad Instat 3.05 (GraphPad Software, USA).

## Results and discussion

**Fig. 1:**
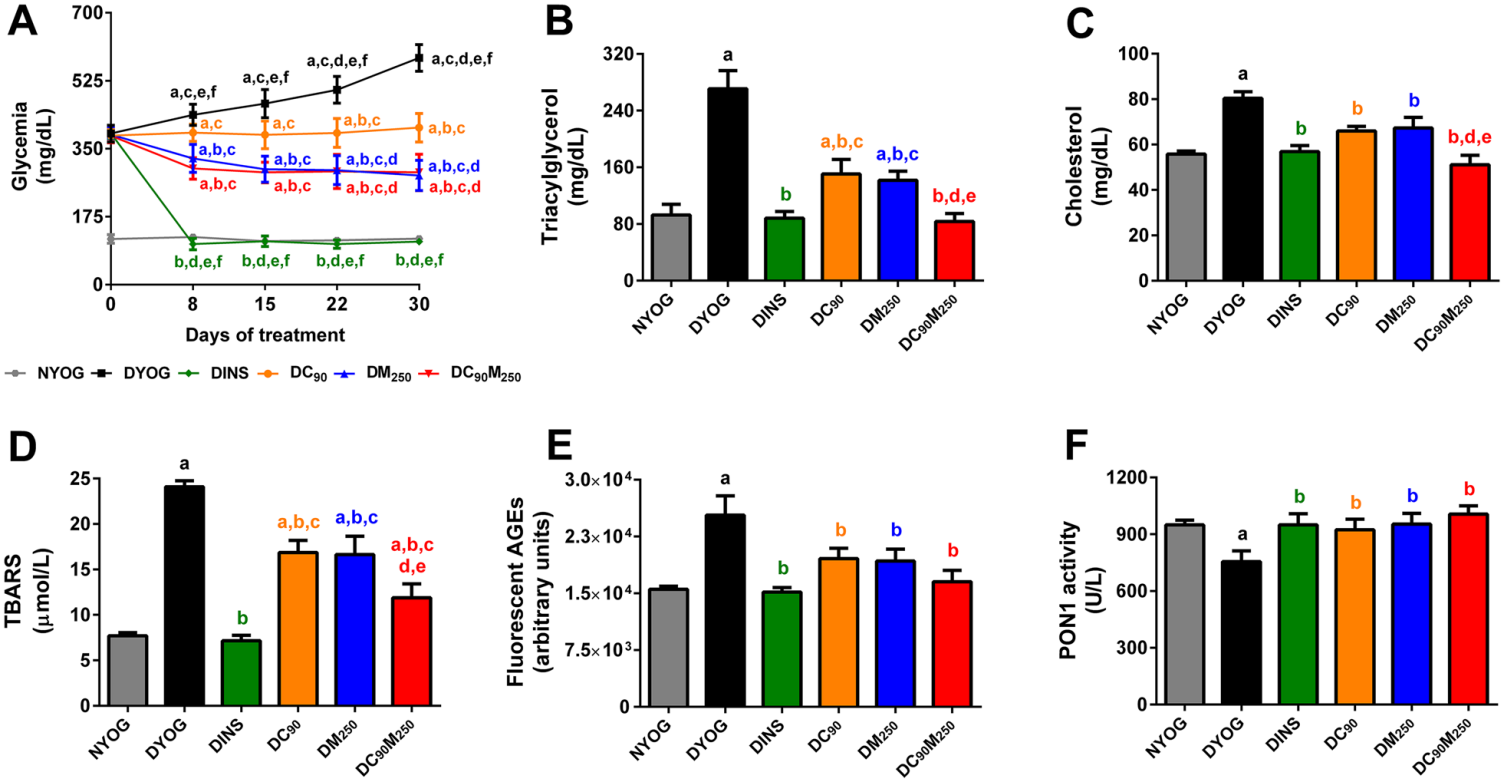
Biochemical and oxidative stress biomarkers of STZ-diabetic rats treated with curcumin alone or combined with metformin. Plasma levels of glucose (**A**), triacylglycerol (**B**), cholesterol (**C**), TBARS (**D**), fluorescent AGEs (**E**), and PON 1 activity (**F**). Values are expressed as mean ± SEM, n = 10. NYOG: normal rats treated with yoghurt; DYOG: diabetic rats treated with yoghurt; DINS: diabetic rats treated with 4 U/day insulin; DC_90_: diabetic rats treated with 90 mg/kg curcumin in yoghurt; DM_250_: diabetic rats treated with 250 mg/kg metformin in yoghurt; DC_90_M_250_: diabetic rats treated with 90 mg/kg curcumin + 250 mg/kg metformin in yoghurt. The differences between groups were considered significant at p < 0.05 and were analyzed using one-way ANOVA followed by Student–Newman–Keuls test. ^a^Differences with NYOG; ^b^differences with DYOG; ^c^differences with DINS; ^d^differences with DC_90_; ^e^differences with DM_250_; ^f^differences with DC_90_DM_250_

Diabetic rats treated with metformin-enriched yoghurt for 30 days had a significant decrease in glycemia levels (Fig. 1A), although the effects of metformin were minor than those of insulin. This reduction in glycemia explain the low levels of glucose excreted in the 24 h urine, as well as the decrease in urinary volume and water ingestion (Table 1). The decrease in glycemia in DM_250_ rats also reflected in minor levels of plasma fluorescent AGEs (end products of protein glycation) (Fig. 1E). The treatment of STZ-diabetic rats with metformin partially restored dyslipidemia, since triacylglycerol (Fig. 1B) and cholesterol levels (Fig. 1C) decreased, but the levels were higher than those in NYOG and DINS rats. The beneficial effects of metformin on lipid profile have been well described [10]. Metformin treatment also decreased the levels of TBARS (biomarker of lipid peroxidation) (Fig. 1D), although in a lesser magnitude than insulin treatment. Improvements in the biomarkers of oxidative damage have been observed in STZ-diabetic rats treated with metformin [11, 12]. Ultimately, the activity of PON 1 increased in diabetic rats treated with metformin-enriched yoghurt (Fig. 1F). PON 1 circulates preferentially in association with HDL, and its antioxidant activity is related to its ability to hydrolyze lipid peroxides within lipoproteins, mainly LDL [13, 14]. Furthermore, Younis et al. [15] observed that HDL has the ability to protect LDL against glycation, and this protection is proportional to the activity of PON 1 found in HDL. In patients newly diagnosed with diabetes, treatment with metformin was able to restore the activity of PON 1 [16].

**Table 1:** Physiological parameters of STZ-diabetic rats treated with curcumin alone or combined with metformin

	Groups											
	NYOG		DYOG		DINS		DC_90_		DM_250_		DC_90_M_250_	
	Day 0	Day 30	Day 0	Day 30	Day 0	Day 30	Day 0	Day 30	Day 0	Day 30	Day 0	Day 30
Body weight (g)	175.80 ± 9.34	332.60 ± 18.82^#^	173.91 ± 3.83	237.08 ± 19.65^a,#^	175.38 ± 4.86	323.13 ± 6.58^b,#^	172.07 ± 2.79	268.42 ± 7.90^a,c,#^	171.06 ± 7.53	267.13 ± 8.97^a,c,#^	173.50 ± 5.08	298.53 ± 9.19^b,#^
Food intake (g/24 h)	20.42 ± 1.59	20.25 ± 2.71	26.31 ± 2.12	41.75 ± 2.06^a,#^	26.83 ± 1.57	22.79 ± 1.05^b^	26.33 ± 1.99	33.21 ± 4.43^a,#^	26.75 ± 1.44	32.64 ± 3.69^a,#^	26.50 ± 1.68	28.00 ± 4.11^b^
Water intake (mL/24 h)	30.17 ± 2.76	26.08 ± 2.28	89.20 ± 12.14	188.33 ± 13.12^a,#^	85.21 ± 13.40	25.78 ± 2.61^b,#^	85.14 ± 7.20	125.67 ± 25.18^a,b,c,#^	86.29 ± 6.73	89.22 ± 5.40^a,b,c^	81.25 ± 7.21	85.31 ± 8.97^a,b,c^
Urinary volume (mL/24 h)	5.42 ± 0.29	16.50 ± 1.31^#^	62.80 ± 10.17	165.17 ± 11.55^a,#^	66.40 ± 9.37	18.50 ± 3.57^b,#^	61.86 ± 6.34	122.67 ± 16.28^a,b,c,#^	63.04 ± 8.67	80.00 ± 11.57^a,b,c^	61.50 ± 10.11	80.60 ± 10.45^a,b,c^
Glycosuria (g/24 h)	nd	nd	1.92 ± 0.53	11.06 ± 0.78^#^	2.52 ± 0.64	0.19 ± 0.041^b,#^	2.58 ± 0.78	2.82 ± 0.67^b,c^	2.21 ± 0.12	2.78 ± 0.13^b,c^	2.31 ± 0.13	2.78 ± 0.19^b,c^

Values are expressed as mean ± SEM, n = 10

The differences between groups were considered significant at p < 0.05 and were analyzed using one-way ANOVA followed by Student–Newman–Keuls test

nd, not determined; NYOG, normal rats treated with yoghurt; DYOG, diabetic rats treated with yoghurt; DINS, diabetic rats treated with 4 U/day insulin; DC_90_, diabetic rats treated with 90 mg/kg curcumin in yoghurt; DM_250_, diabetic rats treated with 250 mg/kg metformin in yoghurt; DC_90_M_250_, diabetic rats treated with 90 mg/kg curcumin + 250 mg/kg metformin in yoghurt

^a^Differences with NYOG; ^b^ differences with DYOG; ^c^ differences with DINS. Differences compared in the same group relative to day 0 were analyzed using the paired Student’s t-test. ^#^ Differences with day 0 (p < 0.05)

A group of diabetic rats (DC_90_) was treated with 90 mg/kg curcumin in yoghurt. The dose of 90 mg/kg curcumin used in the present study was chosen based on a previous study from our laboratory. In this study, Gutierres and collaborators [17] investigated the effects of the treatments of STZ-diabetic rats for 31 days with curcumin-enriched yoghurt at doses of 30 mg/kg, 60 mg/kg, and 90 mg/kg. By monitoring various physiological and biochemical parameters altered in diabetes, authors found many benefits of these treatments, mainly in the glycemic control, and in a dose-dependent response. It must be highlighted that none of these curcumin doses caused toxicity in normal or diabetic rats. In further studies, when 90 mg/kg curcumin was administered in combination with carotenoids, lycopene or bixin [7], or with insulin [6], the effectiveness of curcumin was also observed in STZ-diabetic rats, and these combined treatments were able to add new benefits to combat the complications of diabetes associated with oxidative stress. In the present study, STZ-diabetic rats treated with curcumin-enriched yoghurt for 30 days showed maintenance of the glycemia values throughout the experimental period, in comparison with the progressive increase in the glycemia of DYOG rats, demonstrating the beneficial effects of curcumin on glycemic control (Fig. 1A). This beneficial effect of curcumin helps to explain the decrease in glycosuria, urinary volume, and water intake in DC_90_ rats (Table 1). The antihyperglycemic activity of curcumin can be associated to its potential to prevent inflammation and apoptosis of pancreatic beta cells, improving their function [18], as well as improving insulin responses and glucose tolerance [19]. The minor levels of fluorescent AGEs in the plasma of diabetic rats treated with curcumin-enriched yoghurt (Fig. 1E) can be explained partially by its beneficial effects on glycemia levels, leading to minor AGEs formation. In addition, Sun et al. [20] found that curcumin was able to trap methylglyoxal, a dicarbonyl compound acting as an intermediary in AGEs formation, therefore contributing to our findings of the decreased plasma AGEs levels. The treatment of diabetic rats with curcumin also decreased the plasma levels of TBARS (Fig. 1D). The antioxidant power of curcumin per se [21] may explain its ability to inhibit lipid peroxidation; however, the improvement in glycemia control can also contribute to reduction in the formation of reactive oxygen species (ROS) and therefore decreases oxidative stress in DM. The treatment of diabetic rats with curcumin-enriched yoghurt prevented the reduction in the activity of PON 1 (Fig. 1F), as previously observed [7]. It has been found that curcumin can increase the expression of PON 1 in the liver [22]; however, it cannot be overlooked that PON 1 activity was preserved due to protection against glycation events, since curcumin reduced the markers of glycoxidative stress. Evidence indicated that glycation of PON 1 decreases its activity [23]. Curcumin also improved lipid profile, since the plasma levels of triacylglycerol (Fig. 1B) and cholesterol (Fig. 1C) decreased in DC_90_ rats. The beneficial effects of curcumin on hyperlipidemia have been well described [2, 24]. Considering all these findings, curcumin has appeared as an antioxidant of natural origin with a great potential to oppose the complications of DM.

In a retrospective cohort study with 72,971 participants (patients with type 2 DM in transition to insulin therapy), Xu et al. [25] found that metformin is the most common medication used prior to insulin initiation, in comparison with antidiabetic drugs belonging to other classes. In addition, most patients continued with the metformin therapy after insulin initiation. Moreover, two recent prospective observational studies performed by the global DISCOVER study program found that first-line treatments for patients with type 2 DM were mostly metformin mono-therapy and combinations of metformin with a sulfonylurea. In addition, the most commonly prescribed second-line therapies for type 2 DM were combinations of metformin with a dipeptidyl peptidase-4 inhibitor or metformin with a sulfonylurea [26]. In this sense, the search for novel combined therapies based on natural medicines with the ability to improve the effectiveness of glycemic control promoted by metformin, and to delay or even avoid the transition to insulin therapy is of great interest. In addition, it is known that oxidative stress in DM is often associated with a phenomenon known as “metabolic memory”, where the risk of diabetic complications persists even after glycemic control has been pharmacologically achieved [27], therefore the association of metformin with natural antioxidants that attenuate oxidative stress may be useful in attenuating the long-term complications of DM. Having additional choices beyond combining classical antidiabetic agents, therapeutic options should be personalized to individual patient, combining efficacy, safety, lower costs, and minor risks of long-term complications, co-morbidities and mortality observed in diabetes [28]. In an interesting review by Gupta and collaborators [29], quantitative (pharmacokinetics) and qualitative (pharmacodynamics) interactions were seen in the associations between natural compounds and conventional antidiabetic drugs, including metformin. Many combinations of metformin with natural preparations have shown additive effects, mainly related to the antihyperglycemic potentials. On the other hand, some combinations changed the bioavailability or the renal clearance of metformin, as well as caused hypoglycemia; these findings deserve caution and indicate the need for further investigations.

As far as we know, combinations of metformin with curcumin emphasizing improvements in the control of the symptoms and complications of DM have not yet been studied. In addition to various preclinical studies indicating that curcumin has the potential to ameliorate the symptoms of DM, substantiating clinical studies are also showing promising findings about the efficacy of this phytochemical on glycemic control. A large randomized, double-blinded, placebo-controlled cohort with prediabetic individuals in progression to type 2 DM showed that 9-month treatment with curcumin (six capsules per day, each capsule contained 250 mg curcumin extract) improved the pancreatic beta cell function and various biomarkers related to glycemic control, including fasting glycemia, glycemia after 2 h glucose load, and glycated hemoglobin [30]. In a randomized, placebo-controlled, single blinded, cross-over study, the supplementation with curcumin (tablet containing 180 mg curcumin, one single administration) in healthy individuals, reduced postprandial rise in glucose and insulin serum concentrations [19]. Finally, in a recent review by Rivera-Mancía et al. [31], a large amount of evidence from basic research to clinical studies, was found about the antidiabetic properties of curcumin, with emphasis on its antihyperglycemic, anti-inflammatory, and antioxidant activities. The authors also suggested as future perspectives, the need for advances about the interactions of curcumin with conventional antidiabetic drugs, which may give rise to a new complementary therapy in the management of diabetes. In this regard, the present study demonstrated that the beneficial effects of the combined therapy of metformin and curcumin on diabetes can be achieved via two strategies: (i) the best effects of the isolated treatments were maintained including the reduction in glycosuria and plasma AGEs levels (curcumin and metformin effects), the increase in the activity of PON 1 (curcumin and metformin effects), and the reduction in glycemia (metformin effect); (ii) additive effects were reached, mainly by further decrease in plasma triacylglycerol, cholesterol, and TBARS, in which levels were minor compared to those of isolated treatments. The main benefits achieved by combining metformin and curcumin were the antioxidant potential of this therapy, and the improvement of dyslipidemia. Corroborating our findings, Gutierres et al. [6] recently found additive effects on the reduction of dyslipidemia and plasma lipid peroxidation in STZ-diabetic rats treated with insulin (administered subcutaneously in a minor dose) combined with curcumin-enriched yoghurt (administered orally). Recently, Asadi et al. [32] compared the isolated effects of metformin and curcumin on the attenuation of nephropathy in diabetic rats. The authors observed that both treatments were effective in promoting protection of renal function, but only the treatment with curcumin was able to increase the activities of antioxidant enzymes, and attenuate oxidative stress in kidneys of diabetic rats. Unfortunately, the authors did not investigate the protective potential of the combined therapy. In summary, these findings indicated curcumin as an interesting option to be used in combination with antidiabetic agents to manage the symptoms of diabetes and prevent its long-term complications, mainly those related to oxidative stress.

## Conclusions

Taking into account that combination therapy of current antidiabetic agents with natural antioxidants having beneficial effects on diabetic disturbances might be an interesting strategy, the present study showed the additive effects of curcumin combined with metformin on decreasing dyslipidemia and glycoxidative stress in diabetic rats, in association with the maintenance of metformin effects on decreasing glycemia and the curcumin effects on increasing PON 1 activity. To the best of our knowledge, by targeting not only hyperglycemia, but also oxidative stress and dyslipidemia, this study provides the first evidence about a promising therapeutic strategy by combining curcumin and metformin for the management of DM complications, especially the cardiovascular events. Further research is needed to allow the understanding of the mechanisms of action of this combined therapy that result to the beneficial effects observed in diabetes.

## Abbreviations

AGEs: advanced glycation end products
DM: diabetes mellitus
PON 1: paraoxonase 1
ROS: reactive oxygen species
STZ: streptozotocin
TBARS: thiobarbituric acid reactive substances
